# Wnt signaling activation induces CTCF binding and loop formation at *cis*-regulatory elements of target genes

**DOI:** 10.1101/gr.279684.124

**Published:** 2025-08

**Authors:** Anna Nordin, Chaitali Chakraborty, Mattias Jonasson, Orgena Dano, Gianluca Zambanini, Pierfrancesco Pagella, Silvia Remeseiro, Claudio Cantù

**Affiliations:** 1 Wallenberg Centre for Molecular Medicine, Linköping University, SE-581 83 Linköping, Sweden;; 2 Department of Biomedical and Clinical Sciences, Division of Molecular Medicine and Virology, Faculty of Medicine and Health Sciences, Linköping University, SE-581 83 Linköping, Sweden;; 3 Science for Life Laboratory, Linköping University, SE-581 83 Linköping, Sweden;; 4 Wallenberg Centre for Molecular Medicine, Umeå University, 901 87 Umeå, Sweden;; 5 Department of Medical and Translational Biology, Division of Molecular Medicine, Umeå University, 901 87 Umeå, Sweden

## Abstract

Wnt signaling plays a pivotal role during development and homeostasis. Upon pathway activation, CTNNB1 (also known as beta-catenin) drives the expression of target genes from regulatory regions bound by TCF/LEF transcription factors. Gene regulation, however, entails the interplay between sequence information and 3D genome structure, yet the impact of Wnt signaling on genome structure has been poorly explored. Here, we investigate how Wnt signaling influences CTCF and cohesin, key regulators of 3D genome organization. We identify a series of novel CTCF binding sites that emerge upon Wnt stimulation: CTCF Redistributions Under Wnt (RUW). RUW sites are characterized by CTCF, cohesin, and TCF/LEF occupancy, and are dependent on beta-catenin. Beta-catenin and CTCF colocalize upon pathway activation, and disruption of selected binding sites perturbs target gene regulation. Moreover, Wnt signaling reorganizes the 3D genome as evidenced by genome-wide alterations in CTCF-bound loops. This work reveals a previously unexplored role for CTCF in the regulation of Wnt signaling.

The human body is composed of trillions of cells, most containing an identical genome sequence. The remarkable ability of cells to differentiate into hundreds of specialized cell types is attributed to numerous factors, of which transcriptional regulation is the primary determinant (Jovanovic et al. 2015). Transcriptional regulation, therefore, dictates cell identity, function, and behavior. This complex process depends on the coordinated actions of signaling molecules, receptors, secondary messengers, and transcription factors that work in tandem to form intricate signaling pathways. Yet, these pathways only represent a fraction of the diverse processes that govern cellular behavior. Technological advances have revealed the role of epigenetic modifications and three-dimensional (3D) chromatin structure in transcriptional regulation, adding another layer of complexity to our understanding of cellular behavior (Jerković and Cavalli 2021).

Wnt signaling encompasses three highly conserved signaling pathways that are activated by WNT ligands. Canonical Wnt signaling, referred to as the CTNNB1 (also known as beta-catenin)-dependent pathway, plays a crucial role in cell communication, modulating various cellular responses like proliferation, migration, and differentiation (Nusse and Clevers 2017; Wiese et al. 2018). During development, Wnt/beta-catenin signaling directs early cell fate determination and gastrulation, later driving processes like axis patterning and organogenesis (Grainger and Willert 2018). In adults, Wnt signaling maintains tissue homeostasis by regulating adult stem cells (Reya and Clevers 2005; Barker et al. 2007). Germline mutations that aberrantly activate the Wnt/beta-catenin pathway can lead to developmental disorders, whereas somatic mutations can contribute to the development of cancer (Rim et al. 2022). Colorectal cancer is a well-known example of a Wnt/beta-catenin-driven cancer, with over 80% of cases exhibiting loss of function in the tumor suppressor gene *APC* (Rubinfeld et al. 1993). At the molecular level, the Wnt/beta-catenin signaling pathway is inactive in the absence of WNT ligands. The beta-catenin destruction complex, which incorporates APC and the kinase GSK3, phosphorylates beta-catenin, causing its ubiquitination and subsequent degradation. This results in low levels of free beta-catenin. Upon binding of WNT ligands to cell surface receptors, the destruction complex is recruited to the cell membrane, inactivating it. Beta-catenin then builds up in the cytosol and translocates to the nucleus, where it binds to the TCF/LEF family of four transcription factors and acts as a transcriptional co-activator (Mosimann et al. 2009). Although numerous Wnt/beta-catenin target genes are well-known and seemingly ubiquitous, accumulating evidence is indicating that the nuclear response of Wnt signaling is largely tissue-specific (Söderholm and Cantù 2021). The underlying mechanisms responsible for this specificity are not well understood, representing a crucial area of research for enhancing our comprehension of cell biology and developing therapeutic interventions for Wnt/beta-catenin-driven diseases.

The architecture of the genome plays a crucial role in gene regulation. How DNA is packed into chromatin determines the accessibility of DNA for transcriptional regulators, and how genes are organized in 3D domains further affects interaction between transcriptional complexes, thus influencing the rate of gene expression (Boltsis et al. 2021). During interphase, each chromosome occupies its own territory, and chromatin is further segregated into two main compartments (A and B), which correlate with transcriptional activity (Rowley and Corces 2018). Additionally, at a lower scale, chromatin is organized into topologically associating domains (TADs) which emerge from multiple nested loops by loop extrusion mechanisms (Dixon et al. 2012; Nora et al. 2012; Fudenberg et al. 2016). TADs are, therefore, three-dimensional structures that organize the genome into functional and physical units by bringing together genes and regulatory elements that interact primarily with each other (Dixon et al. 2012; Nora et al. 2012). TADs are thought to form through loop extrusion: as cohesin moves along the chromatin fiber, it traps a loop of chromatin within its ringlike structure until a pair of CCCTC binding factor (CTCF) molecules is reached, which dimerize to anchor and stabilize the loop (Ohlsson et al. 2001; Fudenberg et al. 2016; Davidson et al. 2023). Although TADs are large in size, similar processes also occur at a smaller and more transient scale during chromatin loop formation, often between enhancers and promoters or between regulatory elements (Guo et al. 2015). The binding profiles of CTCF have been extensively studied in various organisms and cell types, and despite differences among species, many CTCF binding sites are highly conserved, indicating their important functional roles (Phillips and Corces 2009; Arzate-Mejıá et al. 2018). Whereas enhancer–promoter looping can occur through other mechanisms, studies have shown that CTCF along with cohesin can either block or facilitate such interactions, with long-range interactions being more dependent on CTCF than short-range interactions (Andrey et al. 2017). Even small changes in CTCF binding patterns can affect local gene regulation during stem cell differentiation (Su et al. 2021), epithelial-to-mesenchymal transition (Essafi et al. 2011), and cancer (Hyle et al. 2019; Chachoua et al. 2022). Studies have shown that high levels of Wnt activation can lead to de novo enhancer–promoter chromatin loops during differentiation (Guo et al. 2021) and that CTCF expression is correlated with that of key Wnt pathway genes in gastric cancer (Liu et al. 2021), but the specific role of CTCF in Wnt signaling, in particular concerning CTCF's ability to regulate the 3D genome, is not well understood. Here, we aimed to explore the potential role of CTCF in the execution of the Wnt signaling transcriptional program.

## Results

### CUT&RUN reveals changes in CTCF and cohesin occupancy upon Wnt signaling activation

**Figure 1.**
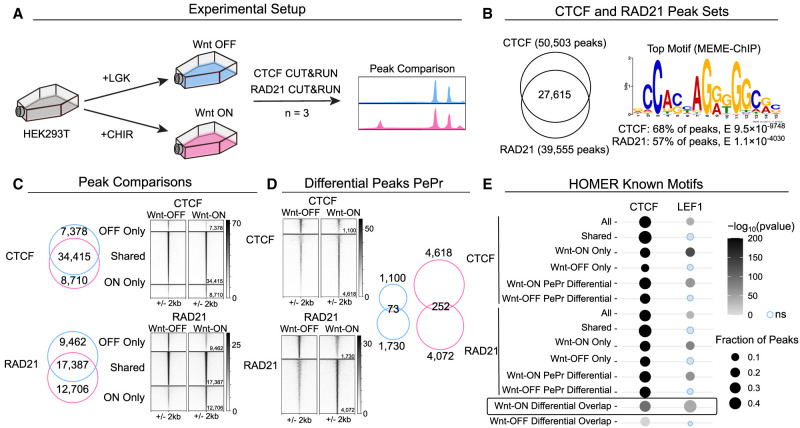
CUT&RUN of CTCF and RAD21 binding in Wnt-OFF versus Wnt-ON. (*A*) Schematic depicting the experimental strategy. LGK was used to induce Wnt-OFF, and CHIR99021 was used to activate Wnt-ON. Three independent biological replicates were performed per condition. (*B*) *Left*: Venn diagram showing overlap of CTCF and RAD21 peaks. *Right*: The top MEME-ChIP motif identified matched CTCF in both CTCF and RAD21 data sets. (*C*) *Left*: Venn diagrams showing overlap of Wnt-OFF (blue) and Wnt-ON (pink) peaks for CTCF (*top*) and RAD21 (*bottom*). *Right*: Signal intensity plots of peak subsets. (*D*) PePr identified differential peaks in Wnt-OFF and Wnt-ON for CTCF and RAD21 and their overlap between CTCF and RAD21 for each condition (*right*). (*E*) Known HOMER motif analysis results for all peak subsets, showing significance and percentage of peaks for CTCF and LEF1 motifs. Only Wnt-ON only subsets display LEF1 enrichment.

In our previous efforts to chart the genome-wide binding profile of beta-catenin, we noticed enrichment for CTCF binding motifs within the beta-catenin peak regions (Doumpas et al. 2019). This finding drove us to hypothesize that Wnt signaling could use CTCF-mediated chromatin organization to regulate target genes. To investigate this, we performed CUT&RUN in HEK293T cells to map CTCF genome-wide binding under Wnt-OFF (i.e., chemical inhibition of PORCN by LGK-974 [LGK]) or Wnt-ON (i.e., inhibition of GSK3 by the small molecule CHIR99021 [CHIR]) conditions, alongside cohesin occupancy by CUT&RUN against the cohesin subunit RAD21 after mild fixation (n = 3 per condition) (Fig. 1A). HEK293T cells display low levels of endogenous Wnt signaling, and therefore the LGK condition resembles closely the DMSO control (Supplemental Fig. S1A), but we chose to use LGK to ensure consistency. We identified a total of 50,503 CTCF peaks (MACS2 *Q* < 0.05) (Zhang et al. 2008), corroborating existing literature which has shown that CTCF binds 40,000–60,000 sites per genome (Arzate-Mejıá et al. 2018), and 39,555 RAD21 peaks, of which 27,615 (70%) overlapped with CTCF (Fig. 1B). The MEME-ChIP Suite (Bailey et al. 2015) identified the top enriched motif in both sets as matching the full CTCF motif, found in 68% of CTCF peaks (E 9.5 × 10^−9748^) and in 57% of RAD21 peaks (E 1.1 × 10^−4030^) (FIMO *P* < 1 × 10^−5^) (Fig 1B; Grant et al. 2011). Quality control metrics for CUT&RUN data sets are included in Supplemental Figure S1B–F.

Out of the 50,503 CTCF peaks, 34,415 (68%) were present in both Wnt-OFF and Wnt-ON conditions, whereas we identified 7378 and 8710 unique peaks for Wnt-OFF and Wnt-ON, respectively (Fig. 1C, top). For RAD21, 17,387 peaks (44%) were common between the two conditions, and 9462 and 12,706 unique to Wnt-OFF and Wnt-ON (Fig. 1C, bottom). Using PePr (Zhang et al. 2014), we found a subset of the peaks for CTCF and RAD21 for which the differential enrichment fulfills FC > 2 and *P* < 0.01 (hereafter referred to as differential peaks) (Fig. 1D). For CTCF, 1100 enriched regions were identified as specific to Wnt-OFF and 4618 regions specific to Wnt-ON conditions (Fig. 1D, top), whereas 1730 Wnt-OFF and 4072 Wnt-ON differential peaks for RAD21 were detected (Fig. 1D, bottom). Overlapping the differential peaks for CTCF and cohesin in each condition, we found that there were 73 Wnt-OFF peaks and 252 Wnt-ON peaks which are differentially bound by both CTCF and cohesin (Fig. 1D). Known motif enrichment analysis using HOMER (Heinz et al. 2010) revealed that CTCF was the top ranked motif in all peak subsets (Fig. 1E). The LEF1 motif was enriched in the Wnt-ON subsets, both for CTCF and RAD21 (Fig. 1E), but never in the Wnt-OFF sets, indicating a role for Wnt signaling in these gained bindings, especially in the subset 252 Wnt-ON peaks differential in both CTCF and cohesin (Fig. 1E).

### Beta-catenin-dependent CTCF Redistributions Under Wnt (RUW)

**Figure 2.**
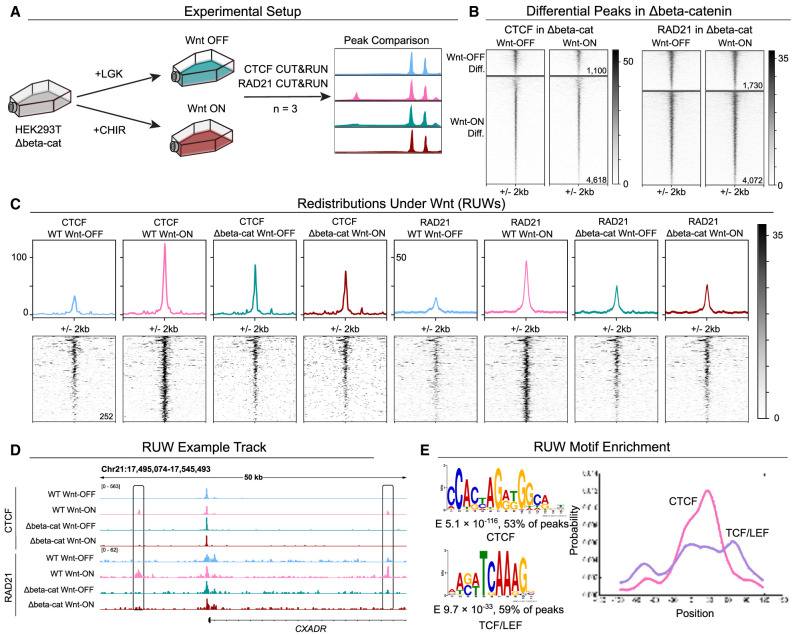
Definition of Redistributions Under Wnt (RUW). (*A*) Schematic depicting the experimental strategy. (*B*) Signal intensity plots of CTCF and RAD21 Δbeta-catenin data sets over peak subsets differential in the WT cells. Δbeta-catenin cells do not show the same signal differences. (*C*) Peak average profiles showing CTCF and RAD21 signal within sites differentially occupied in Wnt-ON by both CTCF and RAD21 (Redistributions Under Wnt). (*D*) Visualization of genomic loci containing RUWs (*CXADR* locus). (*E*) De novo enriched motifs within RUWs matching CTCF (*top*) and TCF/LEF (*bottom*) and their centrality enrichment within the peaks (*right*).

Our chosen method of Wnt signaling induction via stimulation with CHIR99021 is known to activate the pathway via inhibition of GSK3 and thus stabilization of beta-catenin. Because GSK3 inhibition also stabilizes other proteins (Taelman et al. 2010; See et al. 2022), we decided to perform CTCF and RAD21 CUT&RUN also in HEK293T cells lacking beta-catenin (Δbeta-catenin, from Doumpas et al. 2019) to discriminate beta-catenin-dependent and -independent events (Fig. 2A). We focused on our previously identified differential sets of peaks and tested whether they changed upon Wnt activation in the absence of beta-catenin. In Δbeta-catenin cells, signal intensity within these regions does not change upon Wnt activation, indicating that changed occupancy of these sites in WT cells is driven by beta-catenin (Fig. 2B). This was especially visible when looking at the 252 sites differentially bound by both CTCF and RAD21 (Fig. 2C). We decided to focus on these events and termed these 252 sites as CTCF Redistributions Under Wnt (Supplemental Table 1). When normalized and visualized via the Integrative Genome Viewer (IGV) (Robinson et al. 2023), nearby CTCF and RAD21 sites are comparable between cell lines and conditions whereas the RUWs show differences in signal (Fig. 2C,D). Next, we used MEME-ChIP to identify de novo motifs within the RUW sites. RUW sites were most enriched for a motif matching CTCF (E 5.1 × 10^−116^, in 53% of peaks) which was centrally enriched (E 6.1 × 10^−14^), followed by TCF/LEF (E 9.7 × 10^−33^, in 59% of peaks), which was enriched slightly misaligned with respect to the peak center (E 1.3 × 10^−2^) (Fig. 2E). Twenty-five RUW peaks (10%) contained both significant CTCF and TCF/LEF motifs (FIMO *P* < 1 × 10^−5^). The finding that TCF/LEF motifs lay adjacent to those of CTCF, in a close but not overlapping manner, indicated that both factors could simultaneously occupy the same region and strengthened the evidence of these CTCF RUWs being Wnt-driven occurrences.

### RUW sites overlap with characterized Wnt responsive regions

**Figure 3.**
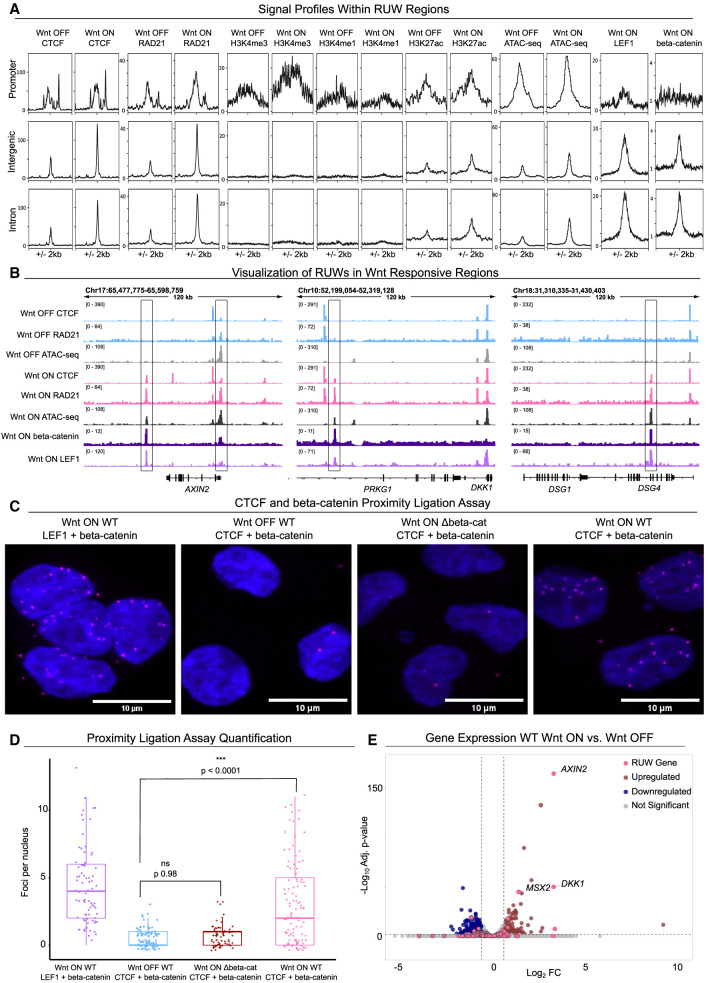
Characterization of RUW regions. (*A*) Signal intensity plots of CTCF, RAD21, H3K4me3, H3K4me1, H3K27ac, ATAC-seq, LEF1 CUT&RUN, and beta-catenin CUT&RUN within subsets of RUW peaks. (*B*) IGV tracks of RUW sites that show Tn5 accessible chromatin only in Wnt-ON, and beta-catenin, and LEF1 binding. (*C*) Representative microscopy images from the proximity ligation assay, showing LEF1 + beta-catenin in Wnt-ON, and CTCF and beta-catenin in Wnt-OFF, Wnt-ON Δbeta-catenin, and Wnt-ON WT. Scale bars 10 µM. (*D*) Quantification of proximity ligation assay (PLA) signal, in foci per nucleus. Wnt-ON WT had significantly more interactions than Wnt-OFF or Wnt-ON Δbeta-catenin. Counted nuclei = 354. (*E*) Volcano plot of differentially expressed genes (Log_2_FC > 0.5, adj. *P* < 0.05) in Wnt-ON versus Wnt-OFF.

We set out to explore the characteristics of Wnt-driven, beta-catenin-dependent RUWs. To this aim, we first annotated them based on their genomic positions. The majority of RUWs were located in introns, followed by intergenic and promoter regions (Supplemental Fig. S2A). Next, we explored RUW regions based on their chromatin characteristics by measuring how they were marked by the histone modifications H3K4me3, H3K4me1, and H3K27ac with CUT&RUN LoV-U (Fig. 3A; Supplemental Fig. S2C). As expected, H3K4me3 signal was mostly found in promoter RUWs, which increased in signal upon Wnt induction, indicating that these promoters become more active. H3K4me1 enrichment within RUWs was generally low, although the signal within promoters seemed to decrease upon Wnt signaling activation. As H3K4me1 is typically depleted from active promoters, we considered it consistent with the increase in H3K4me3 (Sharifi-Zarchi et al. 2017). Many RUWs were decorated with H3K27ac, a marker of active enhancers (Adam et al. 2018): these increased upon Wnt/beta-catenin induction (Fig. 3A; Supplemental Fig. S2C). We recently did a time course study where we mapped chromatin accessibility via ATAC-seq upon Wnt signaling (Pagella et al. 2023). We cross-referenced the RUW sites with this chromatin accessibility data and saw that, whereas most promoters were already accessible and did not change their signal profiles drastically after 24 h of Wnt induction, intron and intergenic RUWs showed gained chromatin accessibility (Fig. 3A, Supplemental Fig. S2B,C). In combination, these data suggested that the RUW sites possess functional activity.

### CTCF and beta-catenin come into physical proximity upon Wnt activation

A comparison of beta-catenin and LEF1 data (Zambanini et al. 2022) and CTCF genome-wide binding profiles revealed—as the motifs indicated—that RUW sites could also be bound by components of the Wnt/beta-catenin nuclear complex: >25% of them were called as beta-catenin peaks and >50% as LEF1 peaks (Fig. 3A, right). The strongest signal for beta-catenin and LEF1 was seen in intron and intergenic RUWs (Fig. 3A, right). An important conclusion from this analysis is that RUW putative enhancers (intron or intergenic sites) show greater changes in CTCF and RAD21 occupancy in comparison to promoter RUWs. Upon Wnt activation, they increase accessibility of chromatin, acquire active marks like H3K27ac, and show binding of LEF1 and beta-catenin. These RUW sites included the notable Wnt targets *AXIN2* and *DKK1* as well as previously unreported direct targets such as *DSG4* (Fig. 3B). The overlapping genomic signal between CTCF and beta-catenin/LEF1 suggested a physical interplay between the Wnt transcriptional complex and CTCF. However, whereas CUT&RUN identifies regions bound by these different factors, it does not distinguish if they ever co-occupy the same locus simultaneously. To test this, we performed proximity ligation assay (PLA), which uses microscopy to detect a signal emerging when two proteins of interest are in close physical proximity (within 40 nm) (Hegazy et al. 2020). Indeed, beta-catenin and CTCF were detected by PLA as proximal on chromatin and only upon Wnt signaling induction (*P* < 0.0001, mean 2.3 foci per nucleus) (Fig. 3C,D; Supplemental Fig. S2D). We performed co-immunoprecipitation to test if we could identify direct binding between LEF1 and CTCF but were unable to detect enrichment over the IgG control (Supplemental Fig. S2E).

### RUW-associated genes include differentially expressed classical Wnt target genes

To further investigate the potential impact of RUWs, we used GREAT (McLean et al. 2010) to assign the RUW peaks to 431 nearby genes (Supplemental Fig. S2F; Supplemental Table 2). STRING (Szklarczyk et al. 2021) mapping revealed a significant degree of interaction within the network (PPI 3.88 × 10^−6^) and Wnt pathway regulators were overrepresented (FDR 0.0092), making up the center of the cluster (Supplemental Fig. S2F). Gene Ontology analysis also revealed enrichment for Wnt pathway regulation (Supplemental Fig. S2G). To explore the potential effect of RUWs on gene expression, we overlapped the RUW genes with expression data in Wnt-ON versus Wnt-OFF (DEG, Log_2_FC > 0.5, adj. *P* < 0.05) (Doumpas et al. 2019). Of the 431 RUW genes, 42 were DEGs (9.7%) consisting of both up- and downregulated genes (20 up, 22 down) (Supplemental Fig. S2H). This includes many of the targets with the highest Log_2_FC, such as *AXIN2, DKK1*, and *MSX2* (Fig. 3E). CTCF itself was not differentially expressed, and thus its expression does not explain the RUWs. To test whether the expression of RUW-associated genes was dependent on beta-catenin and/or TCF/LEF, we analyzed the DEGs upon Wnt induction in Δbeta-catenin and Δ4TCF cells (Doumpas et al. 2019). Whereas most of the upregulated targets were dependent on both beta-catenin and TCF/LEF, the downregulated targets seemed to be mostly independent (Supplemental Fig. S2H), suggesting that GSK3-inhibition-dependent downregulation might occur via different mechanisms.

### Wnt signaling activation leads to large-scale CTCF-mediated genomic reorganization

**Figure 4.**
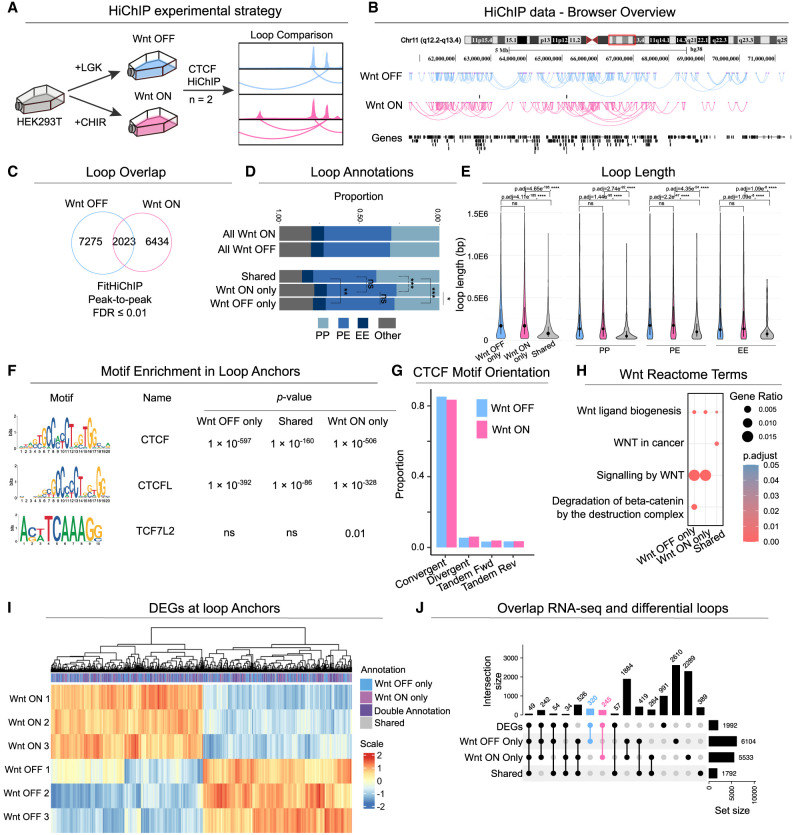
Changes in the CTCF interactome upon Wnt activation. (*A*) Experimental strategy of CTCF HiChIP for Wnt-OFF and Wnt-ON conditions (n = 2). (*B*) Genome browser visualization of significant CTCF HiChIP loops (FitHiChIP FDR ≤ 0.01) in a region of Chromosome 11 in Wnt-OFF and Wnt-ON conditions. (*C*) Loop overlap of significant loops in Wnt-OFF and Wnt-ON conditions, called combining valid pairs across replicates. (*D*) Annotations of all CTCF loops and differential loops (i.e., Wnt-ON/OFF-only, shared) as PP, PE, and EE interactions, where P and E denote promoter and enhancer, respectively. Benjamini–Hochberg adjusted *P*-values are depicted to highlight the significant differences in the proportion of P-P loops and P-E loops across the sets of Wnt-ON-only and Wnt-OFF-only loops, alongside the loops shared between both conditions. (*) *P* < 0.05, (**) *P* < 0.01, (***) *P* < 0.001. (*E*) Loop length analysis for all differential loops (Wnt-ON/OFF-only) (*left*) and differential loops grouped by loop annotation as PP, PE, and EE loops (*right*) (Supplemental Table S5). Statistical testing was performed using a *t*-test, with *P*-values adjusted for multiple comparisons using the Benjamini–Hochberg correction. (*F*) HOMER de novo motif analysis for CTCF, CTCFL, and TCF7L2 within differential loop anchors. (*G*) CTCF motif orientation analysis showing that >85% of loops, in both Wnt-OFF and Wnt-ON conditions, contain convergent CTCF motifs. (*H*) Enrichment dot plot of Wnt-related Reactome pathway terms within differential loops. The *P*.adjust values represent Benjamini–Hochberg corrected *P*-values from Fisher's exact test. (*I*) Heatmap displaying the expression of DEGs (FDR ≤ 0.01) annotated to differential loop anchors. (*J*) UpSet plot depicting the intersections between DEGs (FDR ≤ 0.01) (Doumpas et al. 2019) and genes annotated to the anchors of differential loops.

CTCF and cohesin are known regulators of 3D genome organization, contributing to the formation and maintenance of TADs (Guo et al. 2015). Given that our CUT&RUN data revealed distinct losses and gains of CTCF and cohesin binding following Wnt activation, we wondered whether Wnt perturbation could induce 3D architectural alterations in the genome. Hence, we conducted HiChIP against CTCF for Wnt-OFF and ON conditions (n = 2) (Fig. 4A; Supplemental Fig. S3A–C). To visualize the effect of Wnt activation on 3D genome organization, we displayed CTCF HiChIP data both as loops in the genome browser (Fig. 4B) and as interaction matrices (Supplemental Fig. S3A). Quality control metrics for HiChIP data sets are included in Supplemental Tables 5 and 6 and APA plots in Supplemental Figure S3C,D. To call significant loops, we first processed the sequencing data with HiC-Pro (Servant et al. 2015), merged the data for each condition, and called peak-to-peak loops (i.e., CTCF CUT&RUN peaks at both loop anchors) using FitHiChIP (FDR ≤ 0.01) (Bhattacharyya et al. 2019). Overlap of peak-to-peak loops revealed 7275 loops specific to Wnt-OFF and 6434 specific to Wnt-ON (hereafter referred to as Wnt-ON/OFF-only loops), whereas 2023 loops are shared between both conditions and represent 21.7% of Wnt-OFF and 23.9% of Wnt-ON loops (Fig. 4C; Supplemental Table 5; Supplemental Fig. S4G). Additionally, we performed, in parallel, a replicate analysis, such that we called consensus loops as those present in both replicates for each condition (Supplemental Fig. S4A,G; Supplemental Table 6), showing that our conclusions remain the same independent of the analytical method employed. Altogether, our HiChIP results, therefore, show that, upon Wnt activation, the genome undergoes a prominent topological rewiring.

Genomic annotations of loops show that most CTCF peak-to-peak loops involve promoter–enhancer interactions (P-E loops; ∼42%), whereas a smaller proportion are promoter–promoter (P-P, ∼30%) and enhancer–enhancer (E-E, ∼8%) loops (Fig. 4D; Supplemental Fig. S4B). Whereas promoter–promoter loops were more frequent within the set of shared loops than the Wnt-ON/OFF-only loops, the opposite is observed for promoter–enhancer loops, which were more enriched in the sets of Wnt-ON/OFF-only loops (*P*-adj ≤ 0.05) (Fig. 4D; Supplemental Fig. S4B). Loop length analysis suggests that Wnt activation or inhibition results in the reorganization of long-range chromatin interactions: shared loops are significantly shorter in length compared to unique loops (*P*-adj ≤ 0.001) (Fig. 4E; Supplemental Fig. S4C). These observed changes in chromatin interactions are consistent with the previously reported large-scale changes in 3D genomic structure upon modulation of GSK3 activity, which was shown to decrease the insulation of TADs and increase long-range inter-TAD interactions (Park et al. 2023). Given the central role of canonical Wnt signaling in development, the enrichment of P-E loops might reflect its influence on developmental processes, likely through the formation of rather instructive P-E topologies (Pollex et al. 2024).

A de novo motif search within the anchors of differential and shared loops revealed CTCF and CTCFL as the top two significantly enriched motifs (*Q*-value ≤ 0.05) (Fig. 4F; Supplemental Fig. S4D). The TCF7L2 motif was only enriched in the Wnt-ON-only loop anchors but not in the Wnt-OFF-only or shared loops (*Q*-value ≤ 0.05) (Fig. 4F; Supplemental Fig. S4D). Analyzing the orientation of CTCF motifs within loop anchors, we found >85% of loops with CTCF motifs in convergent orientation (Fig. 4G; Supplemental Fig. S4E), as expected based on previous reports (Rao et al. 2014). Pathway analysis of genes whose promoters are located at the anchors of the differential loops revealed significant enrichment for terms related to Wnt signaling (*P*-adjust ≤ 0.05) (Fig. 4H; Supplemental Fig. S4F). To assess the impact of Wnt-induced topological reorganization on gene expression, we examined whether the expression of genes adjacent to loop anchors is affected. For this purpose, we intersected genes located at the anchors of differential loops with a set of genes that become differentially expressed upon Wnt activation (Doumpas et al. 2019). Nearly half of the differentially expressed genes (DEGs; FDR ≤ 0.01)—47%, 944 genes—are located at the anchors of differential loops (Fig. 4I). More specifically, the promoter regions of 245 DEGs (12%) were located at the anchors of Wnt-ON-only loops, whereas additional 320 DEGs (16%) were linked to loop anchors specific to the Wnt-OFF condition (Fig. 4J). In contrast, only 56 DEGs (3%) were exclusively associated with unchanged loops, shared by both conditions (Fig. 4J). This substantial overlap between differential loops and differential gene expression further supports the idea that Wnt signaling drives topological reorganization that contributes to changes in gene expression.

**Figure 5.**
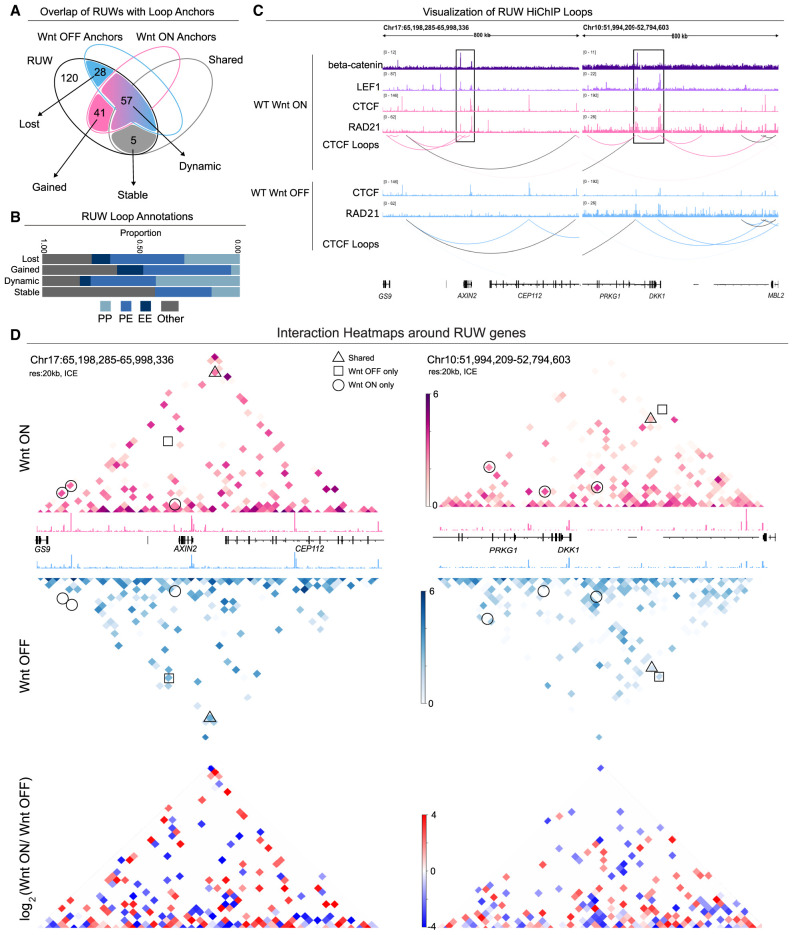
Wnt activation induces changes in CTCF loops around Wnt target genes. (*A*) Venn diagram showing overlap of RUW peaks with CTCF HiChIP loop anchors. (*B*) Annotations of RUW loops as P-P, P-E or E-E loops, where P and E denotes promoter and enhancer, respectively. (*C*) Visualization of Wnt-ON-only CTCF loops associated to RUW peaks at the *AXIN2* and *DKK1* loci. Wnt-ON-only loops are coloured in pink, Wnt-OFF-only loops in blue, and shared loops in black. Wnt-ON-only loops at the *AXIN2* and *DKK1* promoters are highlighted within black boxes. (*D*) CTCF HiChIP interaction matrixes for Wnt-OFF and Wnt-ON (20 kb resolution with ICE normalization, max pixel value 6) alongside corresponding CTCF CUT&RUN tracks. Some differential interactions are highlighted with circles for Wnt-ON-only and squares for Wnt-OFF-only interactions, whereas triangles are shared interactions. The differential matrix (*bottom*) represents log_2_ (Wnt-ON/Wnt-OFF) interactions, such that red indicates enrichment in Wnt-ON and blue enrichment in the Wnt-OFF condition.

Next, we set out to determine whether RUWs were associated with changes in the CTCF-mediated loops. Overall, RUW peaks were mapped to 141 Wnt-OFF-only loops and 119 Wnt-ON-only loops, whereas an additional 97 loops were shared between both conditions (Supplemental Table 3). Moreover, examining the anchors, and upon closer inspection of the intersection between all loop sets and RUW peaks, we found that 131 RUW peaks—representing 52% of all RUWs—coincide with CTCF loop anchors (Fig. 5A). Among these, 41 RUW peaks were found exclusively at Wnt-ON-only anchors and 28 peaks specifically overlapped with Wnt-OFF-only anchors and were annotated as gained and lost anchors, respectively, upon Wnt activation (Fig. 5A). Additionally, 57 anchors overlapping with RUW peaks form different sets of loops, thus representing a dynamic fraction of loops, whereas only five regions were categorized as stable, as they correspond to RUW peaks within shared anchors (Fig. 5A). Notably, gained RUW loops were less enriched for promoter–promoter interactions than lost or dynamic loops and, instead, predominantly involved promoter–enhancer interactions, suggesting a rewiring of the enhancer landscape upon Wnt activation (Fig. 5B). Among these gained loops were those found within the *AXIN2* and *DKK1* loci (Fig. 5C), revealing that RUW sites are involved in new loop formation between enhancers and promoters of classical Wnt target genes. Examination of the interaction matrices (Wnt-ON, Wnt-OFF, Log_2_ Wnt-ON/Wnt-OFF) revealed further alterations in chromatin interactions at the loci of these RUW genes, extending beyond the loops at the promoters (Fig. 5D).

### Perturbation of CTCF binding sites within RUWs affects Wnt target gene upregulation

**Figure 6.**
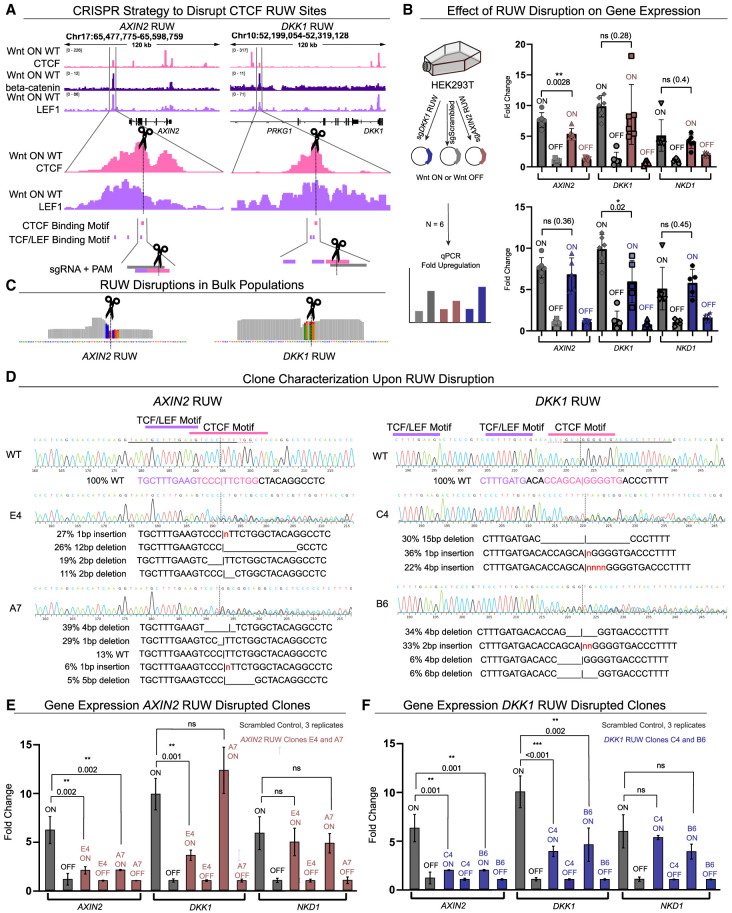
CRISPR-Cas9 disruption of CTCF RUW binding sites. (*A*) CRISPR-Cas9 strategy to mutate RUW CTCF binding sites near *AXIN2* and *DKK1*. sgRNAs were designed to disrupt the CTCF motif. (*B*) Gene expression measurement by qPCR showing that RUW disruption significantly and only affects the upregulation of the corresponding gene for *AXIN2* (*P* = 0.0028) and *DKK1* (*P* = 0.02) compared to a scrambled control. Error bars show mean ± SD among independently transfected populations. (*C*) BAM coverage of RUW sites from sequenced populations with confirmed disruptions in CTCF sites (alteration in PAM sequence + 5 bp upstream). Base pairs with >2% of nonmatching alleles are colored by the representation of each nucleotide. (*D*) Characterization of derived clones, including Sanger sequencing traces and results from ICE deconvolution of alleles, showing two clones for both *AXIN2* and *DKK1* RUWs which have disrupted CTCF binding sites but intact TCF/LEF core motifs. (*E*) qPCR gene expression results of the *AXIN2* RUW clones, which show reduced ability to upregulate *AXIN2.* One clone also shows decreased upregulation of *DKK1*, whereas *NKD1* expression is unaffected. (*F*) qPCR gene expression results of the *DKK1* RUW clones, which show reduced ability to upregulate *DKK1*. Both clones also show reduced upregulation of *AXIN2*, whereas *NKD1* is unaffected. In *E* and *F*, error bars show mean ± SD among technical replicates. Biological replicates (clones) are shown with separate bars. Statistical testing was done with two-tailed *t*-tests.

To test whether CTCF RUWs are functionally implicated in gene expression, we sought to disrupt their CTCF binding motifs. We selected the RUWs annotated to *AXIN2* and *DKK1*, as they are two of the most differentially expressed genes in our analysis and quasi-universal Wnt/beta-catenin targets. The *AXIN2* RUW was located within the characterized enhancer region for the gene (Ramakrishnan et al. 2021), whereas the *DKK1* RUW was located in a putative enhancer region. We identified the exact position of the CTCF and TCF/LEF binding site(s) within these RUW regions and designed CRISPR sgRNAs which would lead Cas9 to disrupt only the core of the CTCF binding motif (Fig. 6A). We transfected cells with Cas9 and either the targeting or a scrambled sgRNA control, and then stimulated to obtain Wnt-OFF and ON conditions (Fig. 6B, left). We first measured gene expression in populations (n = 6 independent wells per condition) and could see that the sgRNA targeting the *AXIN2* RUW led to a significant decrease in upregulation of *AXIN2* upon Wnt activation (*P* = 0.0028) but not *DKK1* nor *NKD1* (Fig. 6B, top panel). Similarly, the sgRNA against the *DKK1* RUW decreased upregulation of only *DKK1* (*P* = 0.02) (Fig. 6B, bottom panel). We confirmed disruption of CTCF binding sites in the populations by NGS sequencing. Detected mutations included indels and sequence alterations which invariably affected CTCF but not TCF/LEF consensus sequences (Fig. 6C). We further validated our results by deriving clones from our mutated populations, isolating two clones each for the *AXIN2* and *DKK1* enhancer regions which contain disrupted CTCF binding sites but intact TCF/LEF binding sites, confirmed via Sanger sequencing followed by ICE deconvolution (Synthego Performance Analysis, ICE Analysis 2019 v3.0; https://github.com/synthego-open/ice) (Fig. 6D; Conant et al. 2022) . We confirmed that, indeed, RUW disruption alters the expression of the corresponding gene in these clones (Fig. 6E,F). These results indicate that CTCF binding to RUW regions contributes to the upregulation of each associated Wnt target gene.

## Discussion

**Figure 7.**
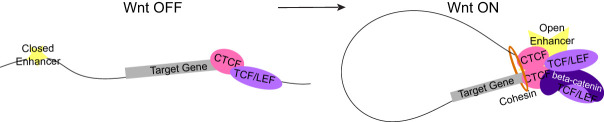
Redistributions Under Wnt model. *Left*: When Wnt is OFF, the chromatin is not accessible at enhancers of Wnt target genes, and transcription is low. CTCF binding is constitutive at a region near the promoter of the gene. *Right*: When Wnt is activated, the enhancer region becomes accessible, and TCF/LEF and beta-catenin bind. CTCF binds the RUW site and, with cohesin, mediates enhancer–promoter looping, which boosts target gene expression.

Enormous efforts have been dedicated to unraveling the complexity of the response to Wnt/beta-catenin signaling activation and deciphering the mechanisms that drive its remarkable tissue-specificity (Söderholm and Cantù 2021). This included the identification of the different components of the Wnt/beta-catenin transcriptional complex (Hecht et al. 2000; Essers et al. 2005; Fiedler et al. 2015; van Tienen et al. 2017) and recently, the varying patterns of beta-catenin genome-wide binding in a time- and context-specific manner (Nakamura et al. 2016; Mukherjee et al. 2020, 2022; Pagella et al. 2023; Ramakrishnan et al. 2023). However, the impact of Wnt/beta-catenin activation on the 3D genome organization, and its potential role in modulating the Wnt-dependent transcriptional output, is still poorly understood. Our establishment of CTCF genome-wide binding patterns across Wnt pathway stimulation and the discovery of the 3D genomic interactions that involve CTCF binding sites lead us to a model where, upon Wnt activation, CTCF is detected at newly accessible Wnt responsive *cis*-regulatory elements. Here, CTCF binds in tandem with beta-catenin and TCF/LEF to increase cohesin stalling and induce the formation of chromatin loops that contribute to the upregulation of a subset of Wnt target genes (Fig. 7). This, to our knowledge, represents a previously unknown mechanism of Wnt target gene regulation.

Although the process by which CTCF is recruited to these sites remains to be solved, our data obtained in Δbeta-catenin cells, where CTCF does not bind RUW sites upon GSK3 inhibition, indicates that the physical presence of beta-catenin is necessary for CTCF redistributions. This would imply that beta-catenin, possibly together with other components of the Wnt transcriptional complex, would access chromatin and recruit CTCF and not vice versa. Although a full time-course of CTCF and cohesin binding upon Wnt activation would be needed to determine the overall time dynamics of RUWs, beta-catenin binding to these sites early (90 min or 4 h) (Pagella et al. 2023) supports the model in which beta-catenin reaches these sites before CTCF. It has previously been reported in the literature that CTCF can bind to or interact with beta-catenin (Chachoua et al. 2022), TCF7 (Shan et al. 2022), and the member of the ChiLS complex LDB1 (Lee et al. 2017), all of which could thus be considered as potential partners in recruiting CTCF to the chromatin. Additionally, it was recently reported that CTCF binding can be context-specific and dependent on the activity of other transcription factors that bind upstream of the CTCF motif and stabilize CTCF binding (Do et al. 2025); we consider this in line with the findings presented in this study.

Recent characterization of the *AXIN2* enhancer—which also corresponds to one of our identified RUWs—has been performed (Ramakrishnan et al. 2021). This study included the introduction of mutations throughout the whole enhancer sequence followed by testing the activity of the mutated variants in a luciferase-dependent Wnt transcriptional reporter. The core of the CTCF binding site that we have identified is located between 288 and 292 bp into the enhancer region characterized by the Cadigan group (see Fig. 5A in Ramakrishnan et al. 2021): mutating this region in their study showed no change in the ability of the enhancer to activate the reporter. Consistently, the in vitro luciferase reporter, although it successfully detected enhancer activity, took the enhancer out of its 3D context of the genome such that one would expect not to find a notable contribution of the 3D chromatin structure to transcriptional activation. Our results would, therefore, imply that only in its native context does this enhancer—and presumably all the others in correspondence to the identified RUWs—need the CTCF binding site to sustain full regulatory activity. This lends credibility to the idea that CTCF, at least in the context of Wnt target gene regulation, is acting as a mediator of genome organization and not as a classical transcription factor (Arzate-Mejıá et al. 2018). Furthermore, the observation that approximately half of the RUW peaks overlap with CTCF-HiChIP loop anchors suggests that Wnt signaling contributes to 3D genome reorganization, potentially through CTCF recruitment.

It has previously been reported that CTCF-mediated chromatin loops are instructive in the formation and maintenance of liquid-liquid phase separated condensates (Lee et al. 2022) and also that beta-catenin becomes a component of these nuclear condensates upon Wnt activation at enhancers of its target genes (Zamudio et al. 2019). This raises the possibility that the RUW-mediated chromatin loops could play a role in the formation of phase-separated transcriptional condensates at Wnt target genes, which might be responsible for driving transcription, a theory to be explored in future research.

In this study, we focused on RUWs that align with the *AXIN2* and *DKK1* genomic loci, as they likely represent the most reliable ubiquitous Wnt target genes (https://web.stanford.edu/group/nusselab/cgi-bin/wnt/target_genes_components). Other targets were not ideal for this scope. As Wnt target genes appear to depend on context (Nakamura et al. 2016; Mukherjee et al. 2020) and time (Pagella et al. 2023), only by exploring other systems and models of Wnt activation can we uncover whether distinct sets of RUWs exist, thereby deciphering whether involvement of CTCF constitutes a mechanism for Wnt signaling to exert its tissue-specific responses. Alternatively, as many RUW genes encode for Wnt pathway negative feedback regulators, it is also possible that CTCF will emerge as a general, and perhaps ubiquitous, facilitator of the negative feedback regulation of the pathway itself. Consistent with our findings, it has been recently reported that CTCF binding near the promoter of genes is correlated with their tissue-specific expression and connection to distal regulatory elements, and Wnt signaling appeared as an enriched GO term for these CTCF-dependent gene sets in multiple tissues (Kubo et al. 2021). Additionally, a new CTCF-AID2 degradation system followed by nascent transcriptional profiling (SLAM seq) identified Wnt signaling as one of the few pathways perturbed after acute degradation of CTCF, implicating CTCF in its regulation (Hyle et al. 2023).

The number of high-confidence beta-catenin-dependent RUWs we focused on was relatively small when compared to the initial ∼8700 CTCF CUT&RUN peaks present only in the Wnt-ON condition, as well as the number of differential cohesin peaks and CTCF HiChIP loops. As the Wnt-ON condition was achieved by CHIR administration, some of these events could be independent of beta-catenin. However, the enrichment of TCF/LEF motifs within the original Wnt-ON-only sets for CTCF and RAD21, as well as within the HiChIP loop anchors, implicates Wnt/beta-catenin in their regulation. The proposition that WNT ligands might act in vivo as selective inhibitors of GSK3 and that stabilization of beta-catenin is only one of the several consequences triggered (Acebron et al. 2014; Huang et al. 2015; Koch et al. 2015) would imply that broad CTCF-mediated 3D genome organization changes will be observed in many other in vitro and in vivo setups. Our study, therefore, underscores the need to investigate the role of CTCF and of the 3D genome organization that follows the response to Wnt activation across all cellular contexts in which this pathway plays a key role.

## Methods

### Cell culture

HEK293T human embryonic kidney cells and Δbeta-catenin cells (generated in Doumpas et al. 2019) were cultured in a 37°C incubator in 5% CO2 and 89% humidity. The culture medium used was high-glucose Dulbecco's Modified Eagle Medium (Gibco, Cat. #41965039) supplemented with 10% bovine calf serum (Sigma-Aldrich, Cat. #1233C) and 1× penicillin-streptomycin (Gibco, Cat. #15140148). Cells were stimulated with 10 µM CHIR99021 (Sigma-Aldrich, Cat. #SML1046) (Wnt-ON) or 1 nM LGK (Selleck Chemicals, Cat. #S7143) (Wnt-OFF), or DMSO (Merck, Cat. #317275) for 24 h.

### CUT&RUN

CUT&RUN for CTCF was performed as described in Skene et al. (2018), with minor modifications as described here. Three independent rounds of experiments were performed for the three biological replicates of each cell line and condition, using 250,000 cells per replicate. Antibodies used included anti-CTCF (abcam, ab70303) and anti-HA (Merck, 05-902R) at 1:100 dilutions. For each sample 40 µL of ConA beads were used, and the digitonin concentration was 0.025% throughout the protocol. After fragment release, DNA was purified with phenol:chloroform:isoamyl alcohol followed by ethanol precipitation.

CUT&RUN LoV-U for H3K4me3, H3K4me1, H3K27ac, and RAD21 was performed according to Zambanini et al. (2022) with minor modifications as described here. Experiments were performed in duplicate for each condition—for RAD21 in triplicate with a mix of both antibodies. Cells for RAD21 samples were fixed with 0.1% formaldehyde in PBS for 10 min, and then quenched with glycine. Starting material used was 250,000 cells per sample and bound to 10 µL magnetic ConA agarose beads. Antibodies used included anti-H3K4me3 (antibodies online, ABIN6971977), anti-H3K4me1 (antibodies online, ABIN3023251), anti-H3K27ac (antibodies online, ABIN2668475), anti-RAD21 (antibodies-online, ABIN2856242), and anti-RAD21 (antibodies-online, ABIN6731038) at 1:100 dilutions. DNA purification of H3K27ac and RAD21 samples was done with phenol:chloroform:isoamyl alcohol followed by ethanol precipitation.

Library preparation was performed according to Zambanini et al. (2022) using the KAPA Hyperprep lit (KAPA Biosystems, Cat. #KK8504) with the following modifications: for CTCF samples, 15 cycles of amplification were performed, whereas LoV-U samples received 13 cycles as previously described. For CTCF samples, size selection was performed postamplification using 2% E-Gel Size Select II Gels (Thermo Fisher Scientific, Cat #G661012), retrieving fragments from 150 to 240 bp (representing 30–120 bp without the adapter sequences). Samples were sequenced with 36-bp paired-end reads on the NextSeq 550 (Illumina) using the Illumina NextSeq 500/550 High Output kit v2.5 (75 cycles) (Illumina, Cat. #20024906).

### CUT&RUN data analysis

Reads were trimmed with bbmap bbduk (version 38.18) (Bushnell et al. 2017) to remove adapter sequences, known artifacts, [AT]_18_, [TA]_18_, and poly(G) and poly(C) repeats. Alignment to the hg38 genome was performed using Bowtie (version 1.0.0) (Langmead et al. 2009) with the options -v 3 -m 1 -X 120 for CTCF and RAD21 and with -X 350 for the histone modification LoV-U samples. SAMtools (version 1.11) (Li et al. 2009) was used for BAM file creation, mate fixing, and deduplication. Bedgraphs were created with BEDTools (version 2.23.0) (Quinlan and Hall 2010) genomecov on paired-end mode. Peaks were called using MACS2 (Zhang et al. 2008) on a merge of three biological replicates against the negative control, using the options –keep-dup all and -f BAMPE on narrowPeak mode with a *Q*-value threshold of <0.05. Differential peaks were identified using PePr (Zhang et al. 2014), using three biological replicates and their negative controls per condition with the settings *P* < 0.01, and then filtered for a FC > 2. Peak sets were subsequently filtered to remove peaks falling within the CUT&RUN suspect list regions (Nordin et al. 2023). Peak overlaps were performed using BEDTools.

For graphs and visualization purposes, replicate BAM files were merged with SAMtools into a single file. deepTools (version 3.5.1-0) (Ramírez et al. 2016) was used to convert BAM files to normalized bigWig files (bamCoverage using -RPGC and -e options), signal intensity plots, and profiles (computeMatrix followed by plotHeatmap). Genome region annotation was done with HOMER (version 4.11) (Heinz et al. 2010) on default settings. Motif analyses were performed with HOMER or the MEME-ChIP Suite (Bailey et al. 2015): Centrimo was run on nonlocal mode. Peak-gene annotation was performed using GREAT (version 4.0.4) (McLean et al. 2010) on default settings. STRING (Szklarczyk et al. 2021) with disconnected nodes removed and SR Plot (https://www.bioinformatics.com.cn/srplot) were used for data visualization.

### Published data integration

RNA-seq data were downloaded from Doumpas et al. (2019) and processed in R (version 4.2.2) (R Core Team 2024) for data visualization. ATAC-seq data were downloaded from Pagella et al. (2023). CUT&RUN data of LEF1 and beta-catenin were downloaded from Zambanini et al. (2022); peaks were called with SEACR (Meers et al. 2019) with a threshold of 0.05 for comparison with RUWs.

### RNA extraction and qPCR

RNA extraction, cDNA conversion, and qPCR were performed according to standard methods; details are in Supplemental Methods. Relative quantification was performed using the method developed by Pfaffl (2001). Statistical testing was done with two-tailed *t*-tests using GraphPad Prism. All primer and sgRNAs are listed in Supplemental Table 4.

### Protein extraction, immunoprecipitation, and western blot

Protein extraction, immunoprecipitation, and western blotting were performed on nuclear lysates according to standard methods; details are in Supplemental Methods. LEF1 was overexpressed using a LEF1-FLAG expression cassette (described in Moparthi et al. 2019) and pulled down using anti-FLAG (Sigma-Aldrich, F1804) and mouse IgG isotype control (Invitrogen, 100400C). Western blotting was performed using anti-beta-catenin (antibodies online, ABIN2855042), or anti-CTCF (abcam, ab70303).

### Proximity ligation assay

Proximity ligation was performed using the Duolink PLA assay kit (Merck, Cat. #DUO92102) according to the manufacturer's guidelines. Antigens were detected using mouse anti-beta-catenin (BD Labs, 610154), rabbit anti-CTCF (abcam, ab70303), and rabbit anti-LEF1 (antibodies online, ABIN1680678) antibodies at 1:200 dilutions. NucBlue (Invitrogen, Cat. #R37606) was used to counterstain for nuclei. The assay was performed in duplicate. Images were acquired using the Zeiss LSM 700 confocal microscope, imaging nuclei and PLA foci on separate channels. Acquisition settings were optimized on the positive control and remained the same for all samples. Images were taken with 40× magnification, taking z-stacks of 10 slices of 1 μm.

Image processing was performed in ImageJ (Schroeder et al. 2021) and Fiji (Schindelin et al. 2012). For visualization, z-stack images were combined into a maximum projection, orange was converted to magenta, and scale bars were added. For quantification, channels were split and converted to 16 bit. Thresholding was applied equally to all images. Analyze particles (>1 μm) was used to count nuclei, whereas PLA foci were counted using find maxima (>100), then foci per nucleus were counted using the ROI measure function. Between 67 and 111 nuclei were counted per condition, for a total of 355 nuclei. Statistical testing was done with two-tailed *t*-tests using GraphPad Prism (version 9.3.0).

### CTCF HiChIP

HiChIP was performed in duplicate in both LGK (Wnt-OFF) and CHIR99021 (Wnt-ON)-treated cell lines using the Arima-HiC^+^ kit (Arima, Cat. #A101020) and according to the guidelines in the Arima-HiChIP user guide for mammalian cells. HEK293T cells were cultured as described above and stimulated for 24 h for Wnt-ON and Wnt-OFF, and ∼5 × 10^6^ cells per line were used to obtain at least 15 µg of input DNA for HiChIP. Cells were fixed in 1% fresh methanol-free formaldehyde for 15 min at room temperature while rotating, and glycine was used to quench the crosslinking reaction (final concentration 125 mM glycine) for 5 min. After two washes with cold PBS, crosslinked cells were harvested in PBS by scraping, pelleted, and stored at −80°C until further usage. In the subsequent steps, crosslinked chromatin was digested with restriction enzymes, end-filled with biotinylated nucleotides, and ligated. Proximally ligated chromatin was then sheared on a Covaris E22O instrument, with the following shearing parameters: shearing time 5 min, PIP 105, duty factor 5, and 200 cycles per burst, to achieve a fragment size range of 200 bp to 800 bp. Chromatin immunoprecipitation was performed with an antibody against CTCF (AbFlex CTCF, Active Motif, Cat. # 91285, Lot 31321002), using 0.5 mg of antibody per mg of sheared chromatin. After biotin enrichment and adapter ligation, immunoprecipitated DNA was subjected to PCR amplification (18 cycles) using the Accel-NGS 2S Plus DNA Library kit (Swift Biosciences, #21024) and indexing kit (Swift Biosciences , #26696), according to the Arima-HiChIP Library Prep user guide. Quality controls for chromatin digestion, ligation, and shearing were conducted through gel electrophoresis on a 1.5% agarose gel, and library profiles were assessed through a Bioanalyzer High Sensitivity DNA Analysis (Agilent, #5067–4626). The HiChIP libraries were sequenced on a NovaSeq 6000 Sequencing System (Illumina) aiming for ∼400 million 150PE reads per library.

### HiChIP analysis

HiChIP analysis was performed as described before (Giambartolomei et al. 2021; Murphy et al. 2024) using HiC-Pro (Servant et al. 2015) and FitHiChIP (Bhattacharyya et al. 2019). FASTQ files were quality-checked with FastQC (https://www.bioinformatics.babraham.ac.uk/projects/fastqc/, 0.11.8). Adapters and low-quality reads (Phred < 33) were removed using cutadapt (v 4.0) (Martin 2011). Mapping to the human genome (GRCh38/hg38) and retrieval of valid interacting fragments was performed using the HiC-Pro software v3.1.0 and setting ligation sites as GATCGATC, GANTGATC, GANTANTC, and GATCANTC, and removing duplicates. HiChIP interaction matrices were generated from valid pairs using HiC-Pro's utility script hicpro2juicebox.sh, and visualized with *Juicer_tools* (v1.22.01) (Durand et al. 2016) and Juicebox (v 1.11.08), as well as HiCPlotter (Akdemir and Chin 2015) for the differential matrices per chromosome or per RUW gene locus by flagging HiCPlotter's compare parameter (-c). Statistically significant loops (FDR ≤ 0.01) were identified using FitHiChIP (v11.0) with a 10-kb bin size and the CTCF CUT&RUN peaks. The parameters set included intype1 for peak-to-peak interactions (i.e., “peak2peak:intype1”, requiring CTCF peaks at both loop anchors) and loop length ranging from minimum 10 kb to maximum 3 Mb (Supplemental Tables 5, 6). Significant loops (FDR ≤ 0.01) were called either by combining valid pairs across replicates for each condition (Figs. 4, 5; Supplemental Table 5) or called on individual replicates prior to obtaining the consensus loops for each condition (Supplemental Fig. S4; Supplemental Table 6). Loop overlap analysis was performed following the approaches by Hu et al. (2023) and Buka et al. (2025), such that two loops were considered overlapping only if both anchors of one loop overlapped by at least 1 bp with the corresponding anchors of the other loop, and using a 10-kb slack extension. All region overlaps were performed using the *GenomicRanges* R package (v1.58.0). Loops specific to the Wnt-ON or Wnt-OFF conditions were labeled as Wnt-ON-only loops or Wnt-OFF-only loops, respectively, and loops which remain invariable across the two conditions were named shared loops. RUW peaks were mapped to differential loop anchors using the *findOverlapsOfPeaks* function from the *GenomicRanges* package (v1.58.0). Aggregate Peak Analysis (APA) was performed using GENOVA (v 1.0.1) to assess the enrichment and quality of chromatin interaction data, both on loops called at 20-kb resolution and on differential loops.

CTCF motif orientation was assessed within a 1-kb window centered on each loop anchor using ENCODE-annotated CTCF motifs from hg38, obtained via the Juicer resource (https://bcm.app.box.com/v/juicerawsmirror/under/opt/juicer/references/genomewide_ctcf_motif_fimo). Loops were classified as convergent when the left anchor contained a CTCF motif on the forward (“+”) strand and the right anchor contained a motif on the reverse (“–”) strand, whereas divergent loops had the opposite configuration. Loops with CTCF motifs on the same strand were labeled tandem forward (both “+”) or tandem reverse (both “−”). The number of loops in each orientation category was quantified using R (v4.4.3) and visualized with *ggplot2* (v3.5.1). De novo motif analysis on loop anchors was performed using HOMER (v4.11.9) across all loops (Wnt-OFF, Wnt-ON) and differential loops (Wnt-OFF only, Wnt-ON only, and shared).

Annotation of all significant and differential loops was done using the GenomicInteractions package in R (1.26.0), and loops were classified as promoter–promoter, promoter–enhancer, enhancer–enhancer or others, setting the criteria for promoter regions <2 kb TSS. Differences in loop length (bp) between differential (Wnt-OFF only, Wnt-ON, and shared loops were assessed using a *t*-test with Benjamini–Hochberg correction with ggpubr (0.6.0). Enrichment analysis for the Wnt signaling pathway was performed using the Reactome database (v3.7). Statistical significance was assessed using Fisher's exact test, and *P*-values were adjusted for multiple testing using the Benjamini–Hochberg FDR correction in R. Genes annotated to the anchors of differential loops were intersected with differentially expressed genes (FDR ≤ 0.01) identified upon Wnt activation (Doumpas et al. 2019), and overlaps were visualized using the *UpSet* function from the R package *ComplexHeatmap* (v2.22.0). Regularized log-transformed (rlog) expression values of differentially expressed genes associated with loop anchors were visualized as a heatmap using the *pheatmap* package (v1.0.12).

### CRISPR-Cas9 RUW disruption

For bulk populations, sgRNAs in Supplemental Table 4 were cloned into the pX330spCas9-HF1 plasmid. pX330-SpCas9-HF1 was a gift from Yuichiro Miyaoka (Addgene, plasmid #108301; http://n2t.net/addgene:108301; RRID:Addgene_108301). For clone generation, sgRNAs were cloned into the LentiCRISPRv2 vector. lentiCRISPR v2 was a gift from Feng Zhang (Addgene, plasmid # 52961; http://n2t.net/addgene:52961; RRID:Addgene_52961). Transfection and lentivirus production details are included in Supplemental Methods.

### Validation of RUW disruptions

For bulk populations, primers were designed as to allow for sequencing of the putative Cas9 cut site within the RUW regions with 36-bp reads. Primer sequences are listed in Supplemental Table 4. RUW regions were amplified from genomic DNA of transfected populations via PCR with the high-fidelity Q5 polymerase (New England Biolabs, Cat. #M0491S), with an annealing temperature of 62° and performing 35 cycles. Correct product size and a single product were confirmed with an agarose gel, then the DNA was purified with bead cleanup to remove primers and contaminants. Amplicons were prepared for sequencing as described above for CUT&RUN samples, with the exception that size selection was performed only to remove adapter dimers. Samples were sequenced to ∼1.5 million raw reads. The Cas9 potential cut site was determined to be within the PAM site plus 5 bp upstream (8 bp total). Reads were trimmed as described above to remove artifacts and then further trimmed to identify amplicons containing WT sequences (containing the WT 8-bp cut site as described above). The full WT cut site was found in 71% of *AXIN2* RUW amplicons and 80% of *DKK1* RUW amplicons. The remaining sequences were aligned to the genome, and visualization of the types of mutations and/or indels was done by viewing the BAM coverage in IGV, highlighting bp with >2% alleles with mutations for the figure, confirming that these occur primarily within the cut site (within the CTCF motif) and do not extend further into other regions of the amplicon (such as the TCF/LEF motif of *AXIN2*).

For clonal populations, genotyping was done by amplification of the RUW site with PCR with the high fidelity Q5 polymerase (New England Biolabs, Cat. #M0491S) using the primers in Supplemental Table 4 and PCR-purified, followed by Sanger sequencing and ICE deconvolution (Synthego Performance Analysis, ICE Analysis 2019 v3.0; https://github.com/synthego-open/ice) (Conant et al. 2022).

## Data access

All raw and processed sequencing data generated in this study have been submitted to ArrayExpress (https://www.ebi.ac.uk/biostudies/arrayexpress) under accession number E-MTAB-13727.

## Supplemental Material

Supplement 1

Supplement 2

Supplement 3

Supplement 4

Supplement 5
