# Supplementary material for: Wnt signaling activation induces CTCF binding and loop formation at cis-regulatory elements of target genes
Source: Genome Res. 2025 Aug;35(8):1701–16. doi: 10.1101/gr.279684.124 (PMC12315713; doi:10.1101/gr.279684.124)
Supplement: Supplement 1 [file Supplemental_Methods.pdf]

RNA Extraction and qPCR

Media was removed and cells were lysed with Qiazol (Qiagen, Cat. #79306). RNA extraction was performed according to manufacturer's guidelines. cDNA conversion was performed using the high-capacity cDNA reverse transcription kit (Thermo Fisher, Cat. #43-668-13) according to manufacturer's guidelines, converting 1-2 ug of RNA per replicate. qPCR was performed using the SYBR Green PCR master mix (Applied Biosystems, Cat. #4309155) in 10 µl reactions on the BioRad CFX96, all reactions were done in technical duplicate or triplicate and the Cq values averaged. Relative quantification was performed using the method developed by Pfaffle to account for primer efficiency (Pfaffl 2001). Housekeeping genes included *GAPDH* and *ACTB*. To calculate fold-change, all relative expression values were compared to the average of the control values for each gene. Statistical testing was done with two-tailed *t*-tests using GraphPad Prism. All primer and sgRNAs are listed in Supp. Table 4.

Protein Extraction

Cells were harvested by scraping, centrifuged, and resuspended in PBS. Nuclear extraction was performed by resuspending in Buffer 1 (10 mM HEPES pH 7.9, 10 mM KCl, 0.1 mM EDTA, 0.1 mM EGTA 1 mM DTT, PMSF) and incubating for 15 min in ice. NP-40 was added to 2% final concentration, samples were vortexed, and then centrifuged. The nuclear pellet was resuspended in Buffer 2 (20 mM HEPES, 400 mM NaCl, 1 mM EDTA, 1 mM EGTA, 1 mM DTT, PMSF) and shaken at 4 degrees for 15 min. Samples were centrifuged at max speed for 5 min and the supernatant taken as nuclear extract.

Immunoprecipitation

Cells were transfected with a LEF1-FLAG expression cassette (described in (Moparthi et al. 2019) using calcium phosphate. After 24 hours, CHIR was given at 10 µM. Nuclear protein was extracted as described above. 30 µl of protein A/G beads (Genscript, L00277) were prepared per sample by washing 3 times in PBS. 20 µl of beads/sample were preincubated ON with 2 µg antibody per sample in 1 mL PBS with 5% BSA. Antibodies used were anti-FLAG (Sigma-Aldrich F1804) and mouse IgG isotype control (Invitrogen, 100400C). After nuclear extraction, lysates were brought to 900 µl and 10 µl of beads were added for pre-clearing and incubated 1 hr at 4 degrees. Pre-clearing beads were discarded.

Antibody coated beads were washed twice in 1 ml PBS, resuspended in 100 µl PBS and then added to lysates and incubated ON at 4 degrees on a rotator. The beads were washed 5 times in PBS 0.05% NP-40 with either 150 mM, 250 mM or 250 mM of NaCl, and then boiled in 30 µl Laemmli 1X for elution.

Western Blot

Samples were loaded on a 10% polyacrylamide gel for SDS-PAGE. Gels were run for 20 min at 90V and then 60 min at 120V. Wet transfer to a nitrocellulose membrane was performed overnight at 4 degrees at 30 V, using Tris-Glycine Ethanol transfer buffer. Membranes were blocked for 1 hr RT in 5% milk in 1X TBST 0.1% Tween. Primary antibody incubation was performed for 1 – 2 hr at RT using anti-beta-catenin (antibodies online, ABIN2855042), or anti-CTCF (abcam, ab70303) antibodies diluted 1:1000. Membranes were washed 4 times for 10 min in TBST. Secondary antibody incubation was performed for 1 hr RT with goat anti-rabbit HRP (Invitrogen, 31460) diluted 1:10,000. Membranes were washed 4 times for 10 min in TBST. Pierce ECL western blotting substrate (Thermo Scientific, 32106) was used for detection, and visualized on the ChemiDoc (Bio-Rad).

CRISPR-Cas9 RUW Disruption

For bulk populations, sgRNAs were cloned into the pX330spCas9-HF1 plasmid. pX330-SpCas9-HF1 was a gift from Yuichiro Miyaoka (Addgene plasmid#108301;http://n2t.net/addgene:108301; RRID:Addgene_108301) Sequences were confirmed via Sanger sequencing. Cells were seeded in 12-well plates 6 hours prior to transfection. Transfections were performed using the calcium phosphate method, with 12 independent transfections performed per sgRNA. 16 hours post-transfection, the cells were washed with PBS and the media was changed. After 24 hours, media was changed to media containing either CHIR99021 or LGK as described above, 6 replicates per sgRNA and condition. After 24 hours, RNA was extracted and qPCR performed as described above.

For clone generation, sgRNAs in Table 1 were cloned into the LentiCRISPRv2 vector. lentiCRISPR v2 was a gift from Feng Zhang (Addgene plasmid # 52961; http://n2t.net/addgene:52961; RRID:Addgene_52961). HEK293T cells were transfected with LentiCRISPRv2 and packaging plasmids, a total of 20 ug of DNA, using calcium phosphate. 24 hours later the media was changed, and

36 hours later media was collected, filtered and polybrene added to 4μg/ml. Virus containing media was added to other HEK293T cells, performing 4 rounds of transduction spaced 12 hours in between each other. 24 hours after the last transduction round, cells were placed under puromycin selection at 2 μg/ml. Non-transduced control cells died under selection after 2 days, and media on transduced cells was replaced with normal media. Cells were diluted and plated in 96-well plates, and observed to ensure growth of clonal populations. Validated clones were plated in 6-well plates, treated with LGK/CHIR, and subjected to RNA extraction, cDNA conversion and qPCR.
