# Supplementary material for: Wnt signaling activation induces CTCF binding and loop formation at cis-regulatory elements of target genes
Source: Genome Res. 2025 Aug;35(8):1701–16. doi: 10.1101/gr.279684.124 (PMC12315713; doi:10.1101/gr.279684.124)
Supplement: Supplement 2 [file Supplemental_Figures.pdf]

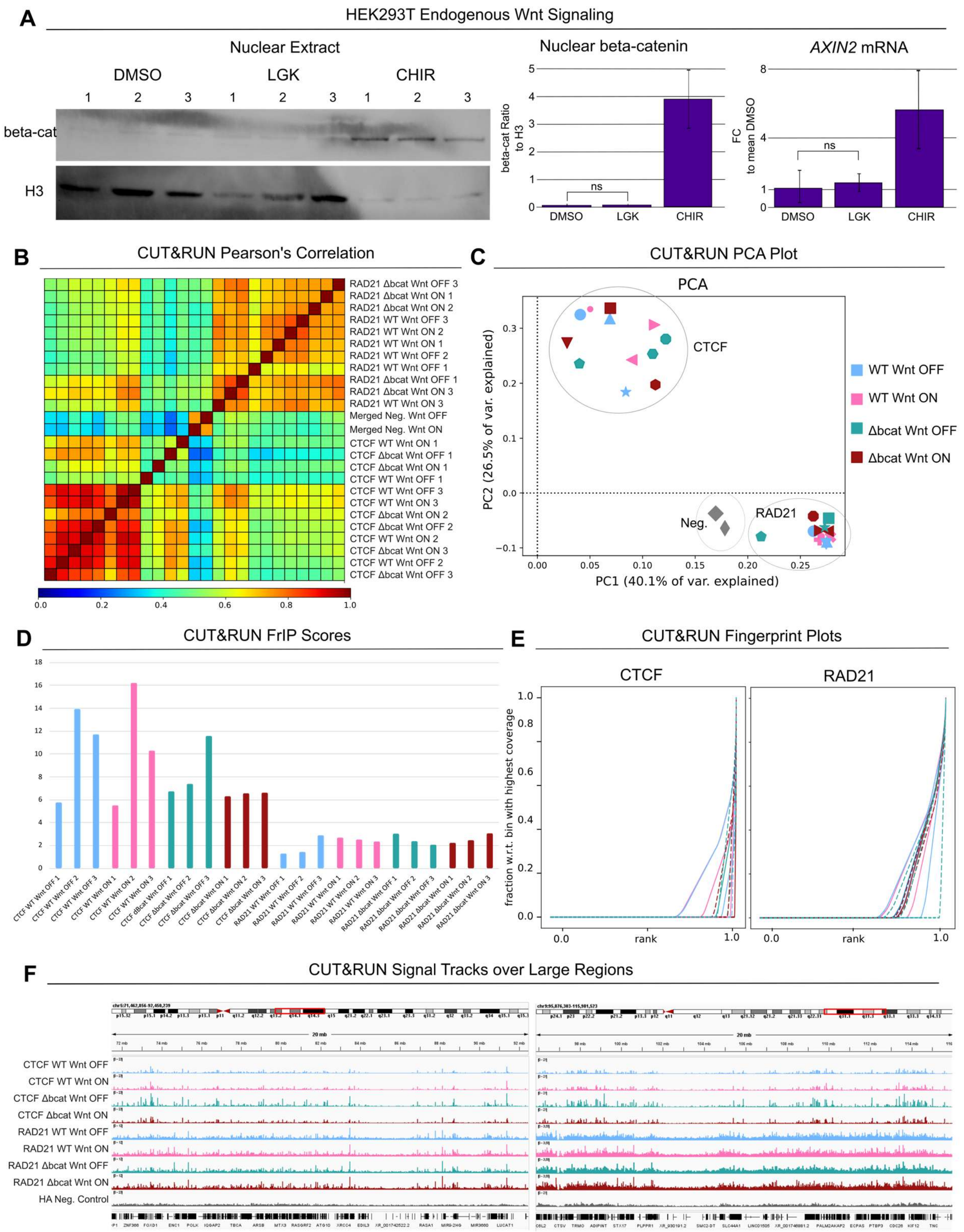

Supp. Fig. S1. A. Left: western blot of nuclear beta-catenin protein levels under DMSO, LGK or CHIR treatment (N = 3). Right: *AXIN2* mRNA expression via qPCR under DMSO, LGK and CHIR. There were no significant differences between DMSO and LGK. **B.** Pearson's correlation calculated from genome wide binning of CTCF and RAD21 CUT&RUN replicates. **C.** PCA plot of CUT&RUN replicates, colored by condition. **D.** Fragments in peaks (FrIP) scores for CTCF and RAD21 CUT&RUN datasets based on fragments within the set of total peaks between WT Wnt-ON and WT Wnt-OFF. **E.** Fingerprint plots of CTCF and RAD21 CUT&RUN datasets. **F.** CTCF, RAD21 and HA negative control CUT&RUN datasets shown in IGV over 20 mb regions, showing consistency between the different conditions and cell lines and between CTCF and RAD21.

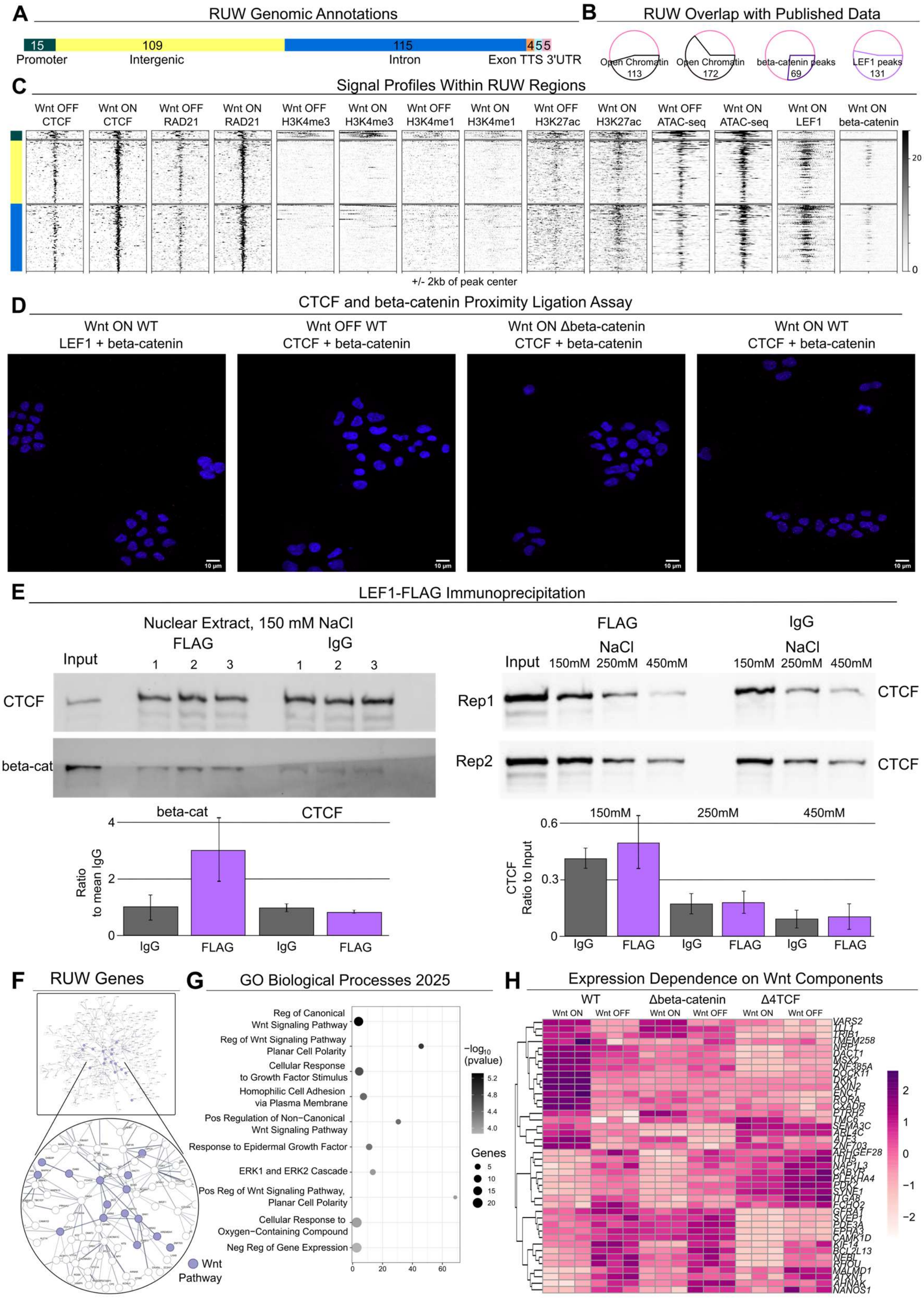

Supp. Fig. S2. A. Annotation of RUWs based on genomic location. **B.** Pie charts of RUW sites called ATAC-seq, beta-catenin and LEF1 peaks. **C.** Signal intensity heatmaps of CTCF, RAD21, histone marks, ATAC-seq, and LEF1 and beta-catenin within RUW peak subsets. **D.** Larger field PLA images. **E.** Co-immunoprecipitation of CTCF with a FLAG-tagged LEF1 protein. beta-catenin was enriched in the pulldown over IgG (left) but CTCF was equal, even with increasing salt stringency (right). **F.** STRING map of RUW associated proteins, bottom: zoom-in on Wnt pathway cluster. **G.** Gene ontology enrichment of RUW genes. **H.** Heatmap of expression of RUW DEGs in WT, Δbeta-catenin and Δ4TCF cells.

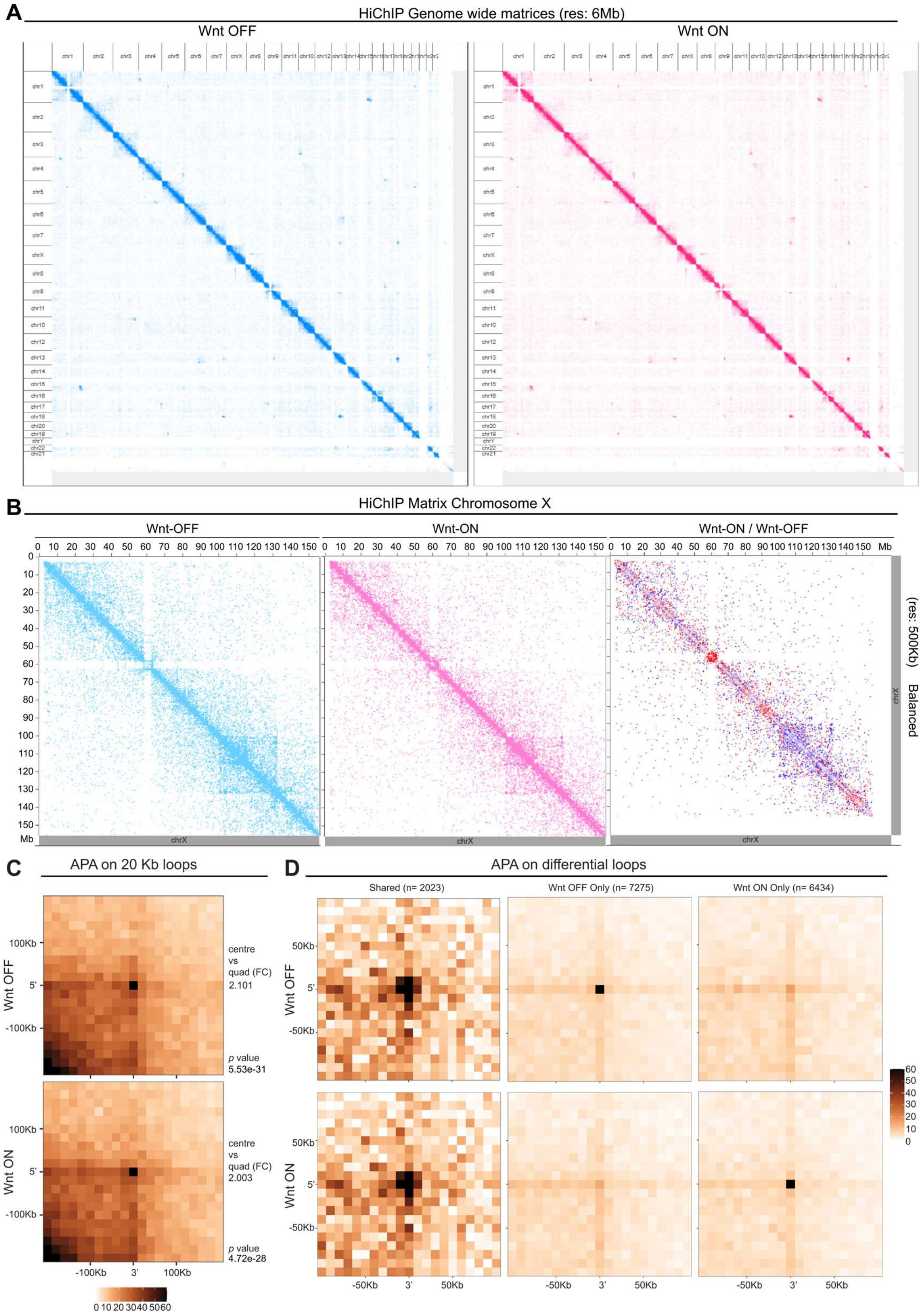

Supp. Fig. S3. A. Genome-wide interaction matrices for CTCF-HiChIP in Wnt-OFF (left, blue) and Wnt-ON (right, pink) conditions at 6 Mb resolution. **B.** HiChIP interaction matrices for Chromosome X in Wnt-OFF (left, blue) and Wnt-ON (center, pink), alongside normalized matrix Wnt-ON/Wnt-OFF (right). Interaction matrices for Wnt-OFF and Wnt-ON were displayed using Juicebox at 500 Kb resolution (pixel=5). **C.** Aggregate Peak Analysis (APA) of statistically significant (FDR $\leq$ 0.01) 20 Kb sample-specific loops using a 20 Kb Hi-C matrix from combined replicate analysis. The fold change and *p* value were calculated with respect to the enrichment of the central pixel to the surrounding 21x21 neighbourhood. **D.** APA analysis on the differential loops showing the specificity of the loops called Wnt-OFF-only and Wnt-ON-only.

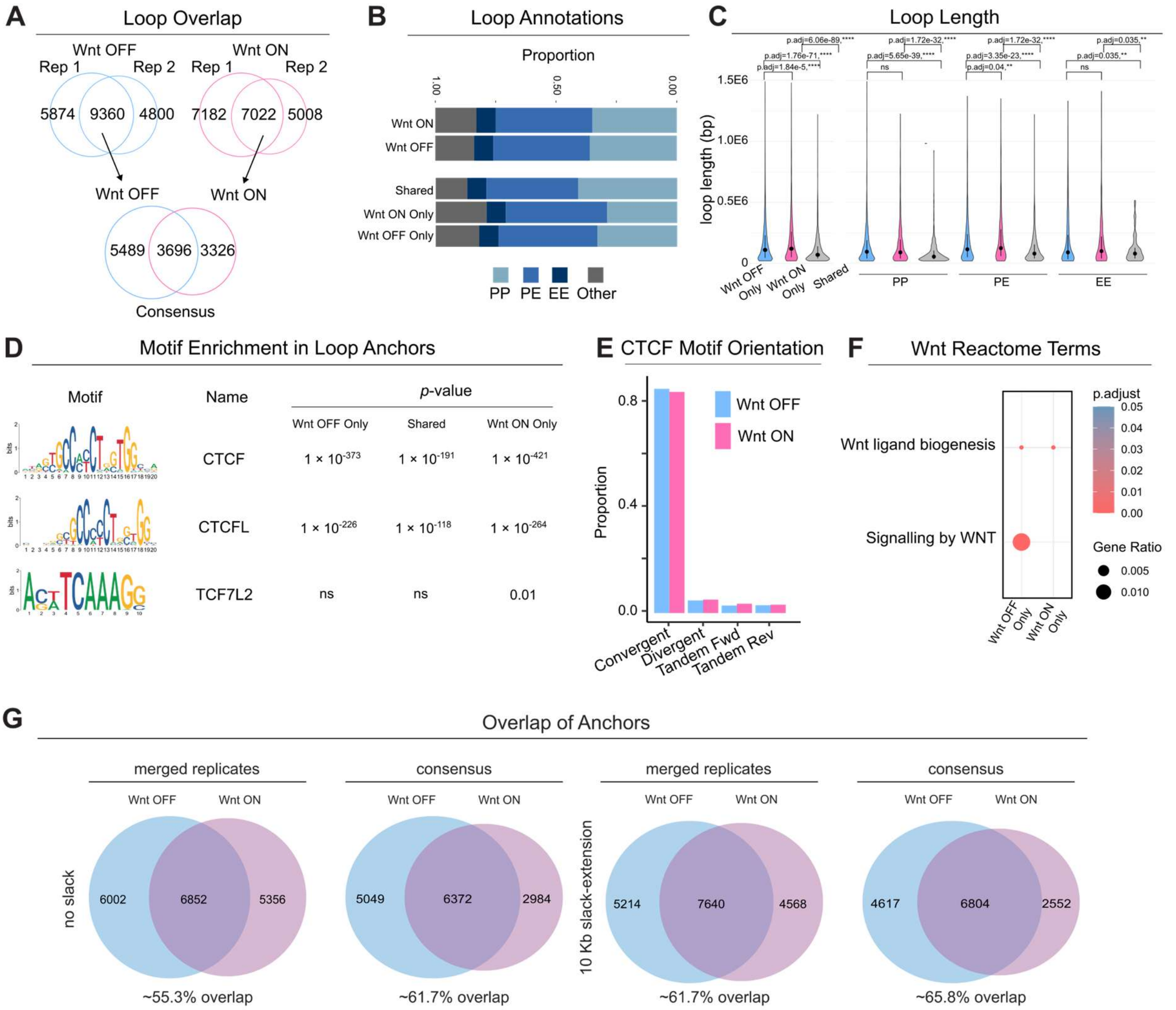

Supp. Fig. S4. HiChIP loops called as consensus loops in the parallel replicate analysis. **A.** Venn diagrams showing overlap of CTCF HiChIP loops across replicates (top; Pearson's correlation $R > 0.99$, $p \leq 0.05$; see Supp. Table 6) and between the sets of consensus loops derived from the replicates for Wnt-OFF and Wnt-ON conditions (bottom). **B.** Genomic annotations of all consensus loops for Wnt-ON and Wnt-OFF, alongside the differential CTCF loops (Wnt-ON-only, Wnt-OFF-only, shared), annotated as PP, PE or EE loops, where P and E denote promoter and enhancer, respectively (for statistical analysis see Supp. Table 6). **C.** Loop length distribution for all consensus differential loops (left), and the subsets of those annotated as PP, PE or EE loops (right). Statistical significance was determined using a *t*-test with Benjamini-Hochberg correction method for multiple testing (see Supplementary Table 6). **D.** HOMER *de novo* motif analysis for CTCF, CTCFL and TCF7L2 within consensus differential loop anchors. **E.** CTCF motif orientation analysis in consensus loops showing that >85% of loops, in both Wnt-OFF and Wnt-ON conditions, contain convergent motifs. **F.** Enrichment dot plot of Wnt-related Reactome pathways within consensus differential loops. **G.** Overlap of loop anchors from significant loops called in Fig. 4 combining valid pairs across replicates ('merged replicates') and consensus loops called in the replicate analysis in Supp. Fig. S4A-F ('consensus'), displayed without no slack (left panels) and with 10 Kb slack extension (right panels).
